# Prevalence of Gastrointestinal Parasites in Blackbuck (*Antelope cervicapra* Linnaeus, 1758) of Blackbuck Conservation Area, Khairapur, Bardia, Nepal

**DOI:** 10.1002/vms3.70884

**Published:** 2026-03-11

**Authors:** Muna Thapa, Janak Raj Subedi

**Affiliations:** ^1^ Central Department of Zoology Tribhuvan University Kirtipur Nepal

**Keywords:** blackbuck, concurrency, gastrointestinal, intensity, prevalence

## Abstract

**Background:**

Blackbuck is a species of antelope native to the Indian subcontinent. This study aimed to investigate the prevalence, diversity, and concurrency of gastrointestinal parasites in the blackbuck population of the Blackbuck Conservation Area in Nepal.

**Methods:**

A total of 150 blackbuck faecal samples were collected and examined using the iodine wet mount and concentration technique.

**Results:**

The findings showed that gastrointestinal parasites were prevalent in 96% of the population, with a higher frequency in females than males. There was no significant statistical association between sex and parasite prevalence across genera (*χ* ^2^ = 9.141, *p* > 0.05). Ten different genera of parasites belonging to protozoa, cestode, trematode, and nematode groups were identified. *Paramphistomum* sp. was the most common, with 55.33% of the cases followed by *Strongyloides* sp. (52%), *Fasciola* sp. (36%), *Haemonchus* sp. (26%), *Moniezia* sp. (24%), *Trichostrongylu*s sp. (21.33%), *Eimeria* sp. (19.33%), *Entamoeba* sp. (15.33%), *Ascaris* sp. (8.67%), and *Trichuris* sp. (7.33%). The study revealed mixed infections ranging from one to six genera in each sample, with triple infections being the most prevalent. Most blackbucks exhibited light infection, while five specific parasite types showed heavy infection levels.

**Conclusions:**

The identification of a significant prevalence and variety of gastrointestinal parasites indicates that parasitism may be an overlooked factor affecting the health of the blackbuck population. This emphasizes the importance of integrating parasite monitoring into wildlife health and conservation efforts.

## Graphical abstract

A. *Eimeria* sp. (31×18 µm, 400x)

B. *Entamoeba* sp. (43×37 µm, 400x)

C. *Moniezia* sp.(63µm, 400x)

D. *Fasciola* sp. (132×78 µm, 400x)

E. *Paramphistomum* sp. (137×81 µm, 400x)

F. *Trichostrongylus* sp. (92×39 µm, 400x)

G. *Ascaris* sp. (48×31µm, 400x)

H. *Haemonchus* sp. (77×46 µm, 400x)

I. *Trichuris* sp. (72×29 µm, 400x)

J. *Strongyloides* sp. (87×56 µm, 400x)

K. *Strongyloides* larva

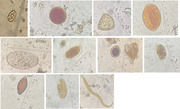

## 1 Introduction

Parasites are ubiquitous in wildlife and an integral part of ecological communities. They are symbionts that coexist in harmony with their hosts (Botzler and Brown 2014; Rose et al. 2014). Intense parasitism can have significant effects on wildlife host populations, altering their reproductive success, fitness, and even their behaviour (Aissa et al. 2021). Exposure to parasites, many of which are exotic and novel to endangered species, has emerged as a major threat to their survival in recent years (Hedrick et al. 2001). Blackbuck (*Antelope cervicapra*) is a graceful gazelle‐like animal. It is regarded as the most attractive member belonging to the family Bovidae, which is classified in the order Artiodactyla and the class Mammalia (Khanal and Chalise 2011; Meena et al. 2017). Species are diurnal ungulates with pronounced sexual dimorphism (Roberts 1997; Sheikh and Molur 2004). Disease can pose a significant threat to endangered species, sometimes causing sudden and unanticipated declines in local abundance (Cleaveland et al. 2002; Muoria et al. 2005). Internal parasites, such as *Haemonchus contortus*, *Trichostrongylus axei*, *Taenia hydatigena*, *Trichuris* sp., *Entamoeba* sp., *Eimeria* sp., *Paramphistomum* sp., *Fasciola* sp., *Moniezia* sp., *Ascaris* sp., *Strongyloides* sp., *Bunostomum* sp., and *Oxyuris* sp., have been identified in the blackbuck population (Chaudhary and Maharjan 2017; Tahir et al. 2021). Several parasites have been found in association with disease and mortality in *Antelope cervicapra*, including *Amphistoma* sp., *Neospora caninum*, *Camelostrongylus mentulatus*, *Strongyloides* sp., *Oesophagostomum* sp., *Strongyle* sp., *Trichostrongylus axei*, *T. probolurus, Toxoplasma gondii*, and *Trichuris* (Goossens et al. 2005; Fagiolini et al. 2010). *Balantidium coli*, *Nematodirus* spp., and *Wenyonella* spp. have been observed in a few species of blackbuck at Bikaner Zoo (Pilania et al. 2014). *Trypanosoma cruzi*‐related ocular lesions and hemorrhagic parasitic conditions, specifically abomasitis and enteritis, are the result of infection by certain types of parasites such as *Haemonchus* spp., *Setaria* spp., and Trichostrongylids (de la Cruz‐Hernández et al. 2015).

Blackbuck Conservation Area (BCA) plays a crucial role in safeguarding Nepal's only wild population of blackbuck. Although conservation activities have generally focused on habitat management, limited attention has been given to disease monitoring and health surveys. This study addresses this gap by providing essential baseline data on the gastrointestinal parasitic burden in blackbuck. Comparing these findings with previous studies will help identify temporal shifts in parasite prevalence and guide better conservation strategies for the long‐term survival of this endangered species in Nepal.

## 2 Materials and Methods

### 2.1 Study Area

**FIGURE 1:**
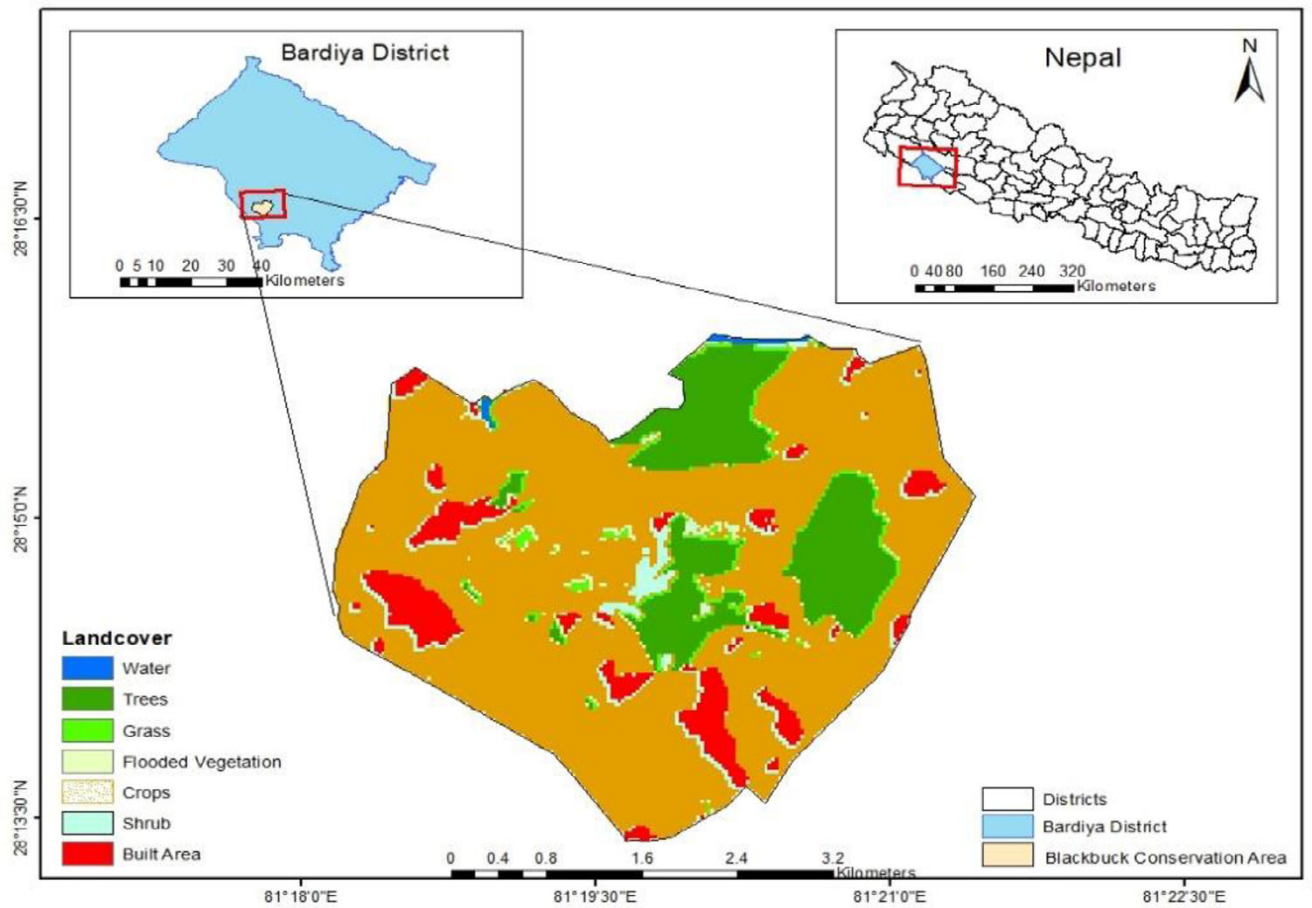
Map illustrating the geographic location of study area.

BCA is in western lowland terai within Gulariya municipality of Bardia district. Its geographical coordinates fall between 20^°^ 07' 54'' and 28^°^ 17' 22'' N latitude and 81^°^ 16' 48'' and 81^°^ 22' 54'' E longitude. In 2009, the Nepal government designated an area of 16.95 km^2^ in Khairapur as BCA, which includes ward number 1, 2, 3 and 4 of the Gulariya municipality. This was the first organized initiative by the government of Nepal to conserve the critically endangered blackbuck. It comprises a core area of 5.27 km^2^ and a community development zone of 11.68 km^2^ (Figure 1).

### 2.2 Sex Identification

To visually identify the sex of blackbuck in a conservation area for faecal sample collection, certain physical characteristics and behaviours were observed. Male blackbuck typically has long, spiral horns that are absent in females and young individuals. Both sexes have distinctive colour patterns, with males displaying a rich dark brown to black upper body, while females have a lighter brown coat. Adult males are generally larger and more robust, with a more muscular neck and shoulder region. Visible genitalia also aided in identification, as males have a prominent scrotum, while females have a urogenital opening. Behaviourally, males exhibit territoriality and aggression, establishing and defending territories and engaging in fights to attract females, while females are more social and form herds with other females and young. When collecting faecal samples from the dominant male blackbuck, it was important to consider their defecation habits, and they often select specific locations within their territory, marked by scraping the ground or creating shallow depressions before defecating.

### 2.3 Sample Collection and Preservation

The study was conducted from 19 August to 18 November of 2022. After determining the blackbuck's primary habitat, fresh faecal samples were collected in sterile vials immediately after the animal defecated in the early morning or at sunset. Samples were collected from each individual blackbuck separately, and they were mostly found in the vicinity of the conservation office and view towers of BCA. The fresh sample was moist, shiny, and dark in colour, usually 1–2 cm in length. Each vial was labelled assigning a reference number. At the onset of the sample collection, the population of blackbuck was 182. However, due to challenges in accurately determining the sex of the fawns, a total of 150 fresh faecal samples were successfully collected from BCA.

Faecal samples were preserved in 2.5% potassium dichromate (K_2_Cr_2_O_7_) solution after collection. It aids in preserving the morphology of protozoan parasites and inhibits the further development of certain helminth eggs and larvae.

### 2.4 Sample Examination

All samples were tested in the Central Department of Zoology laboratory at Tribhuvan University, Kirtipur. The eggs, cysts, oocysts, and larvae of various parasites were identified using morphology and quantitative estimation using Iodine wet mount technique and the concentration method (flotation and sedimentation).

#### 2.4.1 Iodine Wet Mount Technique

This approach is commonly used to detect protozoan eggs/cysts since Iodine makes them visible. A toothpick was used to stir the faecal sample. On a clean glass slide, a solution of 1% Lugol's iodine and a sample of an emulsified toothpick head was placed. Then, a coverslip was delicately kept on top, and smear was thoroughly studied under a microscope (Swift, M4000‐D) with 10× and 40× objective lenses (Zajac and Conboy 2012).

#### 2.4.2 Flotation Concentration Method

Nematode and cestode eggs are lighter than trematode eggs, and this technique is typically used to detect them. They float on a saturated solution of sodium chloride (NaCl). The mixture was prepared by combining about 3 g of faecal sample with 20 mL of distilled water. It was then filtered through a tea strainer after grinding in a mortar. The resulting filtrate solution was transferred into a centrifuge tube (15 mL) and subjected to centrifugation for a duration of 5 min at 2000 rpm in a centrifuge machine (R‐303). Following this, the water was drained from the tube, and saturated NaCl solution was added and centrifuged again. Then, a more saturated NaCl solution was added to generate a convex surface at the top of the tube. A cover slip was placed over the tube for a few minutes, after which it was mounted on a slide and examined under 10× and 40× magnification (Soulsby 2012).

#### 2.4.3 Sedimentation Concentration Method

After examining the floated portion, the saturated salt solution was carefully removed from the test tube, and the sediment content was poured into a watch glass and delicately mixed. One drop was removed from the mixture to prepare a slide. The specimen was stained with a damp mount solution containing iodine. Generally, this technique detects trematode eggs due to their heavy weight and large size (Soulsby 2012).

### 2.5 Parasite Identification and Measurement

To estimate the precise size of parasite oocysts or eggs, the ocular micrometer's reference line (usually the 0 mark) was aligned with a suitable line on the stage micrometer. The perfectly overlapped lines were identified, and the number of divisions on the ocular micrometer occupied by the parasite oocysts or eggs was multiplied by the calibration factor. All measurements were taken using the Erma Inc. ESM‐11 ocular micrometer. The identification of eggs, oocyst, and larvae was done by comparing their morphology, size, and colour (Foreyt 2001; Zajac and Conboy 2012).

### 2.6 Intensity of Parasites

The intensity of parasitic infection was determined with the aid of counting the number of eggs, oocysts, or larvae present within a microscopic field. Infections were classified into four categories: light infection (+), with fewer than two eggs/oocysts/larvae per field; mild infection (++), with two to three per field; moderate infection (+++), with four to five per field; and heavy infection (++++), with six or more per field.

### 2.7 Data Analysis

IBM SPSS Statistics (version 28 IBM Corporation) was used for the analysis after entering all the data into an Excel worksheet (version 2305). Chi‐square (*χ* ^2^) test was used for the statistical analysis of data. In each instance, a statistically significant difference was determined using a 95% confidence interval and threshold of *p*‐value less than 0.05. The prevalence was computed using the formula:

(1) $$\mathrm{Prevalence}=\frac{n}{N}\times 100,$$

 where *n* is the number of positive sample and *N* is the total number of faecal samples examined.

## 3 Results

Among the 150 faecal samples analyzed (63 male and 87 female, *N* = 150), 61 male samples were positive, whereas 83 female samples were infected with one or more gastrointestinal (GI) parasites. Overall, 144 samples were tested positive, indicating a 96% prevalence in blackbuck of BCA. Microscopic examination revealed 10 different genera of GI parasites with four distinct groups, namely protozoa, cestode, trematode, and nematode. Among the identified parasites, *Paramphistomum* sp. (55.33%) had the highest prevalence, while *Trichuris* sp. (7.33%) had the lowest (Table 1). There was no statistically significant relationship between sex and prevalence by genus (*χ* ^2^ = 9.141, *p* = 0.424).

**TABLE 1:** Genera wise prevalence of gastrointestinal parasites in blackbuck.

Serial No.	Identified GI parasites	Number of infected samples		Total prevalence (%)	*χ* ^2^value	*p* value
		Male	Female		9.141	0.424
1.	*Eimeria* sp.	11	18	19.33		
2.	*Entamoeba* sp.	9	14	15.33		
3.	*Moniezia* sp.	15	21	24		
4.	*Fasciola* sp.	23	31	36		
5.	*Paramphistomum* sp.	36	47	55.33		
6.	*Trichostrongylus* sp.	17	15	21.33		
7.	*Ascaris* sp.	5	8	8.67		
8.	*Haemonchus* sp.	23	16	26		
9.	*Trichuris* sp.	4	7	7.33		
10.	*Strongyloides* sp.	26	52	52		

**FIGURE 2:**
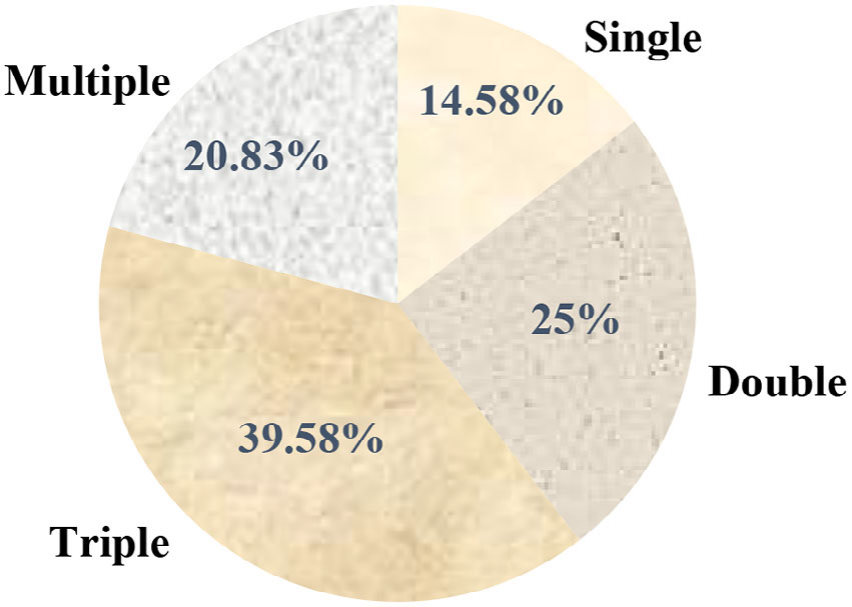
Concurrency of parasitic infection in blackbuck.

The positive samples revealed mixed infection with one to six genera in each sample. The mixed infection was categorized into four types: single, double, triple, and multiple. Out of all the samples, there were 21 samples with a single infection, 36 with two, 57 with three, and 30 with four or more. The highest occurrence was observed in triple infections, which had the most prevalent rate among them (Figure 2). The study showed that there was statistically significant difference in the concurrency of parasitic infection (*χ* ^2^ = 19.5, *p* = 0.0001).

The severity of the parasite infection was evaluated by counting the number of eggs, or oocysts, and larvae that were discovered on each microscope slide. The greatest number of blackbucks was found to be affected by light infection. There were five distinct kinds of parasites that displayed heavy infection (Table 2).

**TABLE 2:** Intensity of parasites in blackbuck.

Parasites	Light (+), *n* (%)	Mild (++), *n* (%)	Moderate (+++), *n* (%)	Heavy (++++), *n* (%)
*Eimeria* sp.	—	—	7 (4.67)	22 (14.67)
*Entamoeba* sp.	18 (12)	5 (3.33)	—	—
*Moniezia* sp.	—	4 (2.67)	13 (8.67)	19 (12.67)
*Fasciola* sp.	17 (11.33)	27 (18)	10 (6.67)	—
*Paramphistomum* sp.	21 (14)	30 (20)	18 (12)	14 (9.33)
*Trichostrongylus* sp.	18 (12)	11 (7.33)	3 (2)	
*Ascaris* sp.	9 (6)	4 (2.67)	—	—
*Haemonchus* sp.	20 (13.33)	12 (8)	5 (3.33)	2 (1.33)
*Trichuris* sp.	11 (7.33)	—	—	—
*Strongyloides* sp.	16 (10.67)	29 (19.33)	12 (8)	21 (14)

## 4 Discussion

The study indicated that parasite prevalence in blackbuck was 96%, which was higher than the findings of previous studies conducted by Pilania et al. (2014) and Chaudhary and Maharjan (2017) that reported prevalence rates of 81.81% and 90%, respectively. Pellet samples from the blackbuck population revealed that 93.33% of the population and 100% of the livestock grazed were infected with at least one form of gastrointestinal parasite. The joint grazing ground of blackbuck and livestock increased the likelihood of parasite and disease transmission (Khanal and Chalise 2011). The absence of deworming practices in the blackbuck population of the conservation area can be a significant contributing factor to the higher prevalence of gastrointestinal parasites.

Host behaviour is crucial in mediating parasite exposure (Ezenwa et al. 2016). In the current study, females had a greater prevalence of gastrointestinal parasites (55.33%) than males (40.67%). This finding was consistent with the results of another study, which indicated that female animals had a higher prevalence of parasite infection than males (Ban 2012). The foraging behaviour of female blackbucks could be a possible explanation for the higher prevalence of parasite infection in this species. Females are frequently found in groups and have a propensity for prolonged grazing. Similarly, out of the total number of antelopes evaluated, 38.5% males and 61.5% females were infected. Physiological changes, such as pregnancy and lactation, may also weaken females' immune systems, making them more susceptible to parasitic infections (Abara et al. 2021).

The findings show that blackbuck harboured several protozoan and helminth species, with *Paramphistomum* sp. (55.33%) and *Strongyloides* sp. (52%) being the most identified parasites. The outcome of this research aligns with the results reported in prior studies carried out by Chaudhary and Maharjan (2017) and Khanal and Chalise (2011) in BCA. In wildlife populations, Singh et al. (2006) recorded that the most detected parasitic infection in herbivores was *Strongyle* spp. Fathima et al. (2017) also noticed *Strongyloides* sp. in the blackbuck population. Blackbucks are in constant contact with the soil, which is often contaminated with helminth eggs or larvae. In addition, *Fasciola* sp. (36%) was also found to be prevalent in the blackbuck population in the findings of this study, which is in line with the findings of Raza et al. (2007). *Fasciola hepatica* was found to be significantly most prevalent (Lashari and Tasawar 2011). In another study, 20.75% infection by *Fasciola* was detected. Several factors contribute to the high prevalence of this parasite, including the presence of suitable intermediate hosts and the ingestion of contaminated water and vegetation (Hossain et al. 2011).

The current study found a prevalence of 21.33% and 26% for *Trichostrongylus* and *Haemonchus*, respectively. This figure was consistent with other research, which reported a prevalence rate ranging from 23.81% to 30% (de la Cruz‐Hernández et al. 2015; Naz et al. 2021). The prevalence may be attributed to species' natural habitat, which is open grasslands or semi‐arid regions. These environments are ideal for the survival and proliferation of these parasites, as they require a warm and humid climate to complete their life cycle.

According to the findings of the present study, *Moniezia* sp. was the only cestode in blackbuck accounting for 24% of overall prevalence, which indicates that this tapeworm is relatively common in this host species. A study by Kar et al. (2007) reported a much lower prevalence of *Moniezia* spp. (3.1%) in goats, which are related hosts. On the other hand, Farooq et al. (2012) and Airee (2018) reported higher prevalence. These findings suggest that the prevalence of *Moniezia* spp. in blackbuck may vary considerably depending on the geographic location and the presence of domestic animal reservoirs. Among the other parasites identified, the prevalence rate was the lowest for *Ascaris* sp. followed by *Trichuris* sp. In a parasitic infection study among captive wild animals, *Trichuris* spp. prevalence showed 19% and ascarid 10% (Mir et al. 2016). The lower prevalence rates found in our study can be related to regional characteristics of parasite distribution and transmission and differences in sample collection.

In this study, *Eimeria* sp. (19.33%) and *Entamoeba* sp. (15.33%) were also detected. One study found coccidia oocysts of the genus *Eimeria* in 2.7% of the specimens examined, while another reported a higher prevalence of 7.14% for *Eimeria* sp. in blackbucks at Bikaner Zoo (Heuschele et al. 1986; Pilania et al. 2014). Another study reported the incidence of *Eimeria* without micropyle in 26%, and Eimeria with micropyle in 15% of the samples examined  (Pun 2018). Furthermore, *Eimeria* sp. was reported in faecal samples from blackbucks at Shuklaphanta National Park (Pant and Joshi 2019). A coprological examination showed an overall prevalence of 22.78% for *Eimeria* sp. (Chouhan et al. 2021). Moreover, in another study, the prevalence was as high as 26.22% for *Entamoeba* sp. (Hassan et al. 2019). The presence of *Entamoeba* sp. and *Eimeria* sp. in the blackbuck population suggests the potential for these parasites to cause disease and highlights the importance of regular monitoring and appropriate management practices to maintain the health of these animals. The variation in prevalence reported across studies may be attributed to differences in factors such as habitat, climate, and management practices.

The current study highlights that blackbuck frequently suffer from multiple parasitic infections, with a high proportion of mixed infections involving several parasite genera. The prevalence of single infections was found to be 14.58%, while double, triple, and multiple infections accounted for 25%, 39.58%, and 20.83% of cases, respectively. This result was consistent with the study by Airee (2018) who found mixed infections, 14.96% for single infections, 34.01% for double infections, 30.61% for triple infections, and 20.4% for multiple infections. Co‐infection might be due to proximity of blackbuck to domestic animals that may serve as a reservoir for a wide range of parasites. Notably, Ghimire and Bhattarai (2019) found that samples examined were concurrently infected with more than two parasites, including up to seven different parasites, indicating a high parasite burden in the population and can have detrimental effects on host mortality. Dhakal et al. (2023) showed that multiple species of animals were infected with at least one type of GI parasites, with varying degrees of concurrent parasitic infections. Grazing in contaminated pastures and lack of effective management strategies could contribute to the acquisition of multiple parasites.

The present study revealed that most of the blackbucks were affected by light parasitic infections. Consistent with these findings, light infections were also reported in the samples examined (Thapa and Maharjan 2015; Pangeni 2021). Furthermore, low‐to‐moderate levels of infestation were observed across all identified parasites (Achhami et al. 2016). It is significant to note that depending on the severity of the infestation, the effects of parasitic illnesses on blackbucks may change.

## 5 Conclusion and Recommendations

This study demonstrates a high prevalence and diversity of gastrointestinal parasites in the blackbuck population. The concurrent infection of multiple parasite genera in a single host further adds to the favourable environment for the coexistence of different genera. These findings underscore the need for improved parasitological surveillance in wildlife conservation efforts.

To mitigate parasitic transmission, it is recommended that grazing of domestic livestock within blackbuck habitats be regulated, as shared resources facilitate cross‐species transmission. Establishing local veterinary facilities near conservation zones could support timely diagnosis and treatment.

## Author Contributions

**Muna Thapa**: Conceptualization; methodology; investigation; data curation; formal analysis; writing – original draft: Janak **Raj Subedi**: Conceptualization; supervision; validation; writing – review & editing; final approval of the version to be published.

## Funding

The authors have nothing to report.

## Ethics Statement

The study was approved by the Department of National Parks and Wildlife Conservation (SN: 079/80‐217) and Blackbuck Conservation Area (SN: 079/080‐42). The animals were not subjected to any experimental infection, and no direct interaction, handling, or disturbance occurred during the sample collection.

## Conflicts of Interest

The authors declare no conflicts of interest.

## Data Availability

The data that support the findings of this study are available from the corresponding author upon reasonable request.
